# Genome-Wide Association Study Reveals the Genetic Architecture of Seed Vigor in Oats

**DOI:** 10.1534/g3.120.401602

**Published:** 2020-10-07

**Authors:** Ching-Ting Huang, Kathy Esvelt Klos, Yung-Fen Huang

**Affiliations:** * Department of Agronomy, National Taiwan University, Taipei, 10617, Taiwan; † Small Grains and Potato Germplasm Research, USDA, ARS, Aberdeen, ID 83210

**Keywords:** Oat (*Avena sativa* L.), seed vigor, genome-wide association

## Abstract

Seed vigor is crucial for crop early establishment in the field and is particularly important for forage crop production. Oat (*Avena sativa* L.) is a nutritious food crop and also a valuable forage crop. However, little is known about the genetics of seed vigor in oats. To investigate seed vigor-related traits and their genetic architecture in oats, we developed an easy-to-implement image-based phenotyping pipeline and applied it to 650 elite oat lines from the Collaborative Oat Research Enterprise (CORE). Root number, root surface area, and shoot length were measured in two replicates. Variables such as growth rate were derived. Using a genome-wide association (GWA) approach, we identified 34 and 16 unique loci associated with root traits and shoot traits, respectively, which corresponded to 41 and 16 unique SNPs at a false discovery rate < 0.1. Nine root-associated loci were organized into four sets of homeologous regions, while nine shoot-associated loci were organized into three sets of homeologous regions. The context sequences of five trait-associated markers matched to the sequences of rice, *Brachypodium* and maize (E-value < 10^−10^), including three markers matched to known gene models with potential involvement in seed vigor. These were a glucuronosyltransferase, a mitochondrial carrier protein domain containing protein, and an iron-sulfur cluster protein. This study presents the first GWA study on oat seed vigor and data of this study can provide guidelines and foundation for further investigations.

Cultivated oat (*Avena sativa* L.) is the seventh most important cereal crop in the world in terms of production and cropping area (FAOSTAT 2017). Oat is mostly known for the benefits to human health of its dietary fiber, beta-D-glucan (Butt *et al.* 2008). Oat is also a good forage crop because of its high biomass, protein content, and digestible fiber (Coblentz *et al.* 2018; Coblentz *et al.* 2012; Contreras-Govea and Albrecht 2006). As forage, oats are grown in a wide range of environments, from temperate regions to the tropics (Suttie and Reynolds 2004). However, oat studies related to forage production in sub-tropic or tropic setup are rare.

Early plant establishment of forage is crucial for subsequent biomass production. Good establishment implies a rapid development of ground cover which can not only lower soil evaporation and facilitate water use efficiency (López-Castañeda and Richards 1994; Rebolledo *et al.* 2015), but also make crops more competitive to weeds (Zhao *et al.* 2006). Plants with higher seed vigor can germinate rapidly and have the ability to establish quickly at early developmental stages (Finch-Savage and Bassel 2016; Ellis 1992).

Seed vigor is a complex concept which encompasses a number of aspects from seed germination to seedling growth, such as rate of germination, uniformity of germination, rate of seedling growth, and uniformity of seedling growth (Ellis 1992). Several factors impact on seed vigor which can be categorized to either intrinsic or external. Intrinsic factors are related to the organism, including its genetic constitution, stage of maturity at harvest, seed size, and seed weight. External factors are those related to the environment, such as the soil type, temperature, water availability that the mother plant encountered, as well as the environmental factors throughout the post-harvest stages. Seed ages and deteriorates during post-harvest storage, causing a decline in vigor. Three key seed vigor traits have been identified as necessary for a good establishment across a wide range of seedbed conditions: the seed must germinate rapidly, have rapid initial downward growth, and have a high potential for upward shoot growth in soil of increasing impedance (Finch-Savage *et al.* 2010).

In oat, seed vigor has been investigated through seed coating treatments (Peltonen-Sainio *et al.* 2006); fungicide treatments and environmental factors encountered by the mother plant (Oliveira *et al.* 2014); the relationships among thermal time, embryo size and other vigor parameters (López-Castañeda *et al.* 1996); and the contribution of genotypes, seed sizes and osmotic potentials to seed vigor (Willenborg *et al.* 2005). However, these studies used only small samples (two to five genotypes), and the differences among genotypes, although statistically significant, were small (Willenborg *et al.* 2005). On the other hand, information on the genetic architecture of oat seed vigor is lacking, unlike other cereal crops, such as rice and wheat, for which genetic studies on seed vigor were available and informed that seedling vigor-related traits were controlled by several loci of moderate to minor effect (Borjas *et al.* 2016; Landjeva *et al.* 2009; Redoña and Mackill 1996; Spielmeyer *et al.* 2007; Sun *et al.* 2007; Wang *et al.* 2018; Zhang *et al.* 2005)

For genetic studies, it is necessary to phenotype a sufficient number of diverse genotypes. Therefore, efficient and appropriate phenotyping methods are needed. Root development is difficult to access and root phenotyping methods are generally destructive, so that the time-series measures on an individual plant, necessary to estimate root growth rate, are not possible. Germination pouches allow a direct observation of roots during growth, and can serve as a good tool to investigate root traits. Compared to other growing systems, like agar or paper roll methods, using germination pouches to phenotype root traits requires much less space and is easy to implement. Root phenotyping is time-consuming, therefore, software for root system analysis, such as RootReader2D (Clark *et al.* 2013), SmartRoot (Lobet *et al.* 2011), or WinRhizo (Arsenault *et al.* 1995) have been developed to investigate in detail the root system. However, some of this software is expensive, and automatic identification of complex root systems has been problematic to achieve. Therefore, an accurate and efficient method to analyze a large number of images would help in the investigation of seed vigor across genotypes.

Genome-wide association (GWA) is a method to investigate the genetic architecture of traits of interest using available germplasm. It takes advantage of ancestral recombination events and can provide higher resolution compared to traditional quantitative trait locus (QTL) mapping using bi-parental populations (Zhu *et al.* 2008). Using GWA, several novel genomic regions associated with coleoptile response of submerged rice plants, as well as 10 genomic regions that were co-localized with published QTL were identified (Hsu and Tung 2015). Campbell *et al.* (2017), applying GWA on a rice diversity panel of *ca*. 360 accessions, have identified *OsGA2ox7*, a gibberellic acid catabolic gene which may regulate early vigor at the tillering stage of rice (Campbell *et al.* 2017). Using GWA, *qTIPS-11*, a QTL that contributes to the lateral root number of rice seedlings, was identified and proven to harbor one causal gene for lateral root number differences using a combination of haplotype analysis, expression assessment, and transgenic approaches. (Wang *et al.* 2018).

As an allo-hexaploid (2n = 6x = 42) with a large genome (12.5 Mbp) (Yan *et al.* 2016b), the development of genomic tools in cultivated oats has been relatively slow and late compared to rice. Meanwhile, thanks to the community effort of oat workers, high-throughput marker systems have been recently made available, such as Illumina 6K gene chip (Tinker *et al.* 2014) or genotyping-by-sequencing (GBS) (Huang *et al.* 2014). These two high throughput genotyping platforms have been widely used by the oat community for genetic studies as well as for breeding applications (Chaffin *et al.* 2016; Esvelt Klos *et al.* 2016; Kebede *et al.* 2019; Sunstrum *et al.* 2019; Yan *et al.* 2016a; Yan *et al.* 2019; Bjørnstad *et al.* 2017; Carlson *et al.* 2019; Tumino *et al.* 2017; Tumino *et al.* 2016). Thanks to the high throughput genotyping platforms, oat GWA was made possible (Bekele *et al.* 2018; Bjørnstad *et al.* 2017; Carlson *et al.* 2019; Esvelt Klos *et al.* 2016; Klos *et al.* 2017; Montilla-Bascón *et al.* 2015; Tumino *et al.* 2017; Winkler *et al.* 2016) even before the recent release of the draft oat genome sequence (*Avena sativa* – OT3098 v1, PepsiCo, https://wheat.pw.usda.gov/GG3/graingenes_downloads/oat-ot3098-pepsico; June 23, 2020). Indeed, the robust consensus map (Chaffin *et al.* 2016) has allowed the anchoring of markers, both from the 6K gene chip or from the GBS approach where the SNPs were called based on alignment between reads where the reference genome sequence was not required (Bekele *et al.* 2018; Lu *et al.* 2013). The development of the high-throughput markers was part of the Collaborative Oat Research Enterprise (CORE), which has also defined a set of germplasm representative of the cultivated oat diversity (Esvelt Klos *et al.* 2016). The CORE panel consisted of 652 oat varieties or breeding lines from major oat breeding programs across North America and Europe. The panel has been genotyped using both 6K gene chip and GBS which revealed the weak population structure within the elite oat germplasm, with a rough distinction of two sub-populations: spring oats *vs.* southern oats, as well as a quick linkage disequilibrium (LD) decay, where pairwise *r^2^* = 0.10 corresponded to an average pairwise distance of 0.44 cM within the whole panel or 0.71 and 2.64 cM within spring and southern oat, respectively (Esvelt Klos *et al.* 2016). GWA has been applied to the CORE panel to identify trait-associated markers for agronomic and disease-resistance traits (Bjørnstad *et al.* 2017; Esvelt Klos *et al.* 2016; Klos *et al.* 2017).

Given the importance of seed vigor for crop establishment, the knowledge gap in the genetics of oat seed vigor, and the available materials provided by the CORE, the objectives of the present study were (i) to develop an image-based phenotyping pipeline for seed vigor; (ii) to characterize the phenotypic variation of seed vigor-related traits within elite oat lines under a temperature regime close to sub-tropic and tropic setup; and (iii) to characterize the genetic architecture of oat seed vigor-related traits using a GWA approach.

## Materials and Methods

### Plant materials

The 650 lines of the Collaborative Oat Research Enterprise (CORE) (Esvelt Klos *et al.* 2016) were evaluated for this study, including 103 lines comprising a world-diversity panel (WDP), 421 lines nominated from spring oat breeding programs, and 126 lines nominated from southern oat breeding programs. Six cultivars Lamont, Ajay, CDC Dancer, Swan, Mountain-1, and NTU-selection No.1 were used as check varieties for batch effect adjustment.

### Experimental design and plant growth condition

Oat lines were grown at two locations as independent replicates, one in the Department of Agronomy, National Taiwan University (Taipei) and the other in the USDA-ARS Small Grain and Potato Germplasm Research (Aberdeen). We used the augmented design (Federer 1956) to accommodate the large sample size while controlling for possible batch-to-batch environmental variability. For each replicate, lines were allocated without replication into eight and twelve batches in Taipei and Aberdeen, respectively, while the six check varieties were assayed with each batch to estimate batch effect. The evaluations in Taipei were conducted from July to October 2018, with 118 to 156 oat lines per batch. The evaluations conducted in Aberdeen were conducted from July 2018 to March 2019 with 48 to 144 lines per batch (see Supplementary Figure S1 for an illustration of experimental design). For each replicate of each line, 10 to 12 plump seeds were manually selected, rinsed in 1% sodium hypochlorite solution for 3 min, and washed three times with RO water. After cleaning, seeds were soaked in RO water for 10 to 12 hr at 25° before being planted in CYG germination pouches (13 cm × 15 cm; Mega international, MN, USA). Nine plump seeds of similar size were evenly and carefully placed with embryos downward in perforations of the germination paper. To support the germination pouches and to facilitate further image taking, a plastic board was placed behind the germination paper in each pouch. Water was added into each pouch after seed set and about 45 pouches were placed in a plastic basin (30 cm × 21 cm × 10.5 cm) with 4 cm tall water. The bottom right corner of the germination pouch was cut prior to seed set to maintain the humidity of the germination paper through capillary action. All pouches were then transferred into a growth chamber at 25° without light till image acquisition.

### Image-based seed vigor phenotyping

#### Image acquisition:

A digital camera (Nikon D3400, Nikon Inc., Tokyo, Japan for the camera used in Taipei and Canon, EOS Rebel XSi, Melville, New York, U.S.A., for the one used in Aberdeen) was set at a height of 42 cm using a copy stand (Kaiser Fototechnik, Buchen, Germany for Taipei and Polaroid MP-4 Land Camera 4401, Polaroid Corp., Cambridge, MA, U.S.A. for Aberdeen). Pictures were taken at day 3, 4, and 5 after setting the seeds into the pouch (day 0). At each photo-taking, germination paper was carefully moved out from the pouch with the help of the plastic board. The camera was set at ISO 400, 22 mm focal length, shutter speed at 1/13^th^ second, and aperture of F/5.6 using manual focus.

#### Image analysis – general principle:

**Figure 1:**
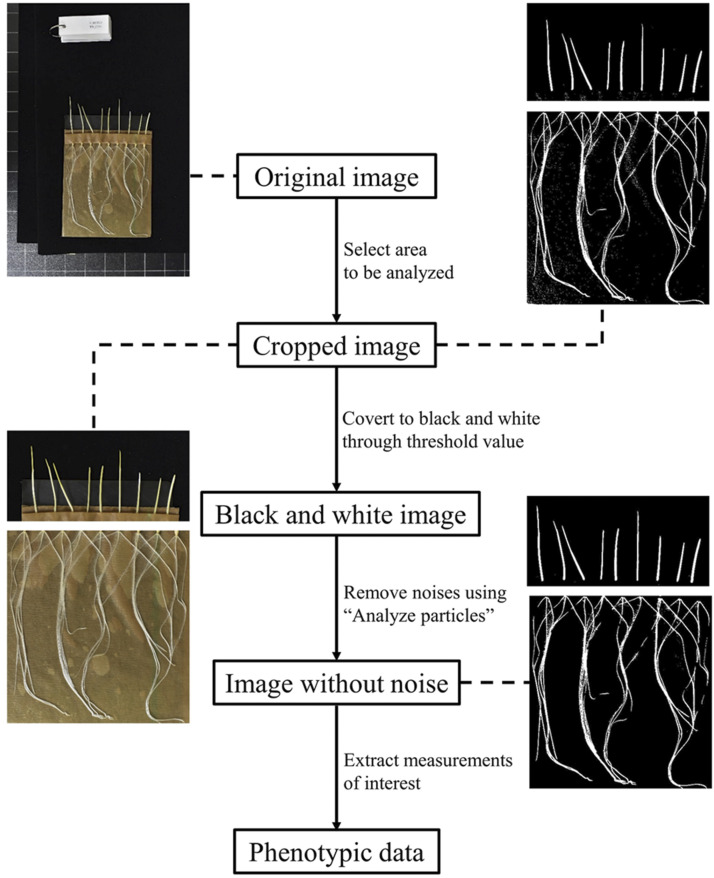
Seed vigor image-based phenotyping pipeline.

Image analysis was conducted using Fiji (Schindelin *et al.* 2012), a distribution of ImageJ (Schneider *et al.* 2012). Fiji has a graphical user interface with the ability to perform image treatment in batch using user-defined commands. Image analysis was performed through three main steps (Figure 1): i) the area to be analyzed was cropped and converted into a black-and-white image using a threshold value, ii) noise was removed, and iii) measurements of interest were extracted. Details of the image analyses of different traits and the threshold value is provided below. Pixels were converted into standard distance units using grids on the copy stand of known size. For images taken in Taipei, 450 pixels equal 30 mm and for those taken in the Aberdeen, 222 pixels equal to 25.4 mm. All images were visually inspected and images were discarded from subsequent analyses if the test line show poor germination (< 5 seed germinated) or poor growth.

#### Image analysis–root traits:

Images were first cropped to keep the area occupied by roots. The cropped images were then split into green, red, and blue channels using “Split channels” in Fiji. The blue channel was retained for its better separation of roots from the background. To extract the root surface area (white to light gray) from the background (dark gray), a threshold value was set empirically at approximate 1.7 times of median gray value of the image and was slightly adjusted within each batch to fully capture the root surface area. Background noise was removed with the help of the “Analyze Particles” function in Fiji: particles with size between 0 and 300 pixels were defined as noise removed; noise with particle size larger than 300 pixels were manually removed after visual inspection. “Create selection” and “Measure” were further used to select and measure root surface area. For each image, the total number of roots and the number of germinated seeds were counted through visual inspection. Other derived traits are listed in Table 1.

**Table 1:** Trait designations and descriptions

Trait name	Acronym	Description (units)	Definition
Original trait			
Root number	RN	Average number of roots	Total root number/number of germinated seeds
Root surface area	RSA_*i*	Root surface area measured at day *i* (mm^2^)	
Shoot length	SL_*i*	Shoot length measured at day *i* (mm)	
Derived trait			
Average root surface area	AVRSA	Root surface area at day 5 divided by root number (mm^2^)	RSA_5/RN
Root growth rate	RG_*i*	Difference of root surface area between two subsequent time points (mm^2^·day^-1^)	RSA_*i* – RSA_*i*-1
Root relative growth rate	RRGR_*i*	Root growth rate divided by the root surface area at the earlier time point (day^-1^)	(RG_*i*)/RSA_*i*-1
Shoot growth rate	SG_*i*	Difference of shoot length between two subsequent time points (mm·day^-1^)	SL_*i* - SL_*i*-1
Shoot relative growth rate	SRGR_*i*	Shoot growth rate divided by the shoot length at the earlier time point (day^-1^)	(SG_*i*)/SG_*i*-1

#### Image analysis–shoot traits:

The shoot part of the image was cropped from the original image and converted to gray-scale using the “8-bit” function. To distinguish shoot from background, a threshold value was set empirically as 1.4 times of median gray value and was slightly adjusted within each batch. Images were visually inspected and noises were manually removed. Shoot was identified using “Analyze Particles” where particle size was set from 300 to infinity pixels. Because most shoots grew upright, we used “Feret diameter”, an option in “Set measurement” to measure the shoot length. All traits investigated in this study are listed in Table 1.

### Phenotypic data cleaning

Lines showing poor growth were removed prior to GWA. “Good” or “poor” growth of each line was defined empirically using two sets of parameters. One set of parameters was root or shoot growth rate between day 4 and day 5 (RG_5 and SG_5, respectively). For root traits, lines were discarded if its RG_5 was smaller than 7 and 4 mm^2^ day^-1^ in Taipei and Aberdeen, respectively. For shoot traits, a line was discarded if its SG_5 was less than 12 mm per day and 4 mm day^-1^ for lines assayed in Taipei and Aberdeen, respectively. The second set of parameters was related to the image quality. For root traits, all images were visually inspected and classified into four categories: category 1 indicated the best quality where all roots were captured using the image analysis method and category 4 indicated the worst quality where less than half of the roots were captured. Only images of categories 1 and 2 were retained for subsequent root-related analyses. For shoot traits, values of average shoot length may be biased by variable emergence of shoots. Therefore, a line was discarded from further analysis if more than one shoot or three shoots were emerged at day 5 for Taipei and Aberdeen, respectively.

### Genotypic data

Genotypic data consisted of 22,767 single nucleotide polymorphism (SNP) markers, among which 4,561 were acquired using the Illumina iSelect 6K chip array and 18,206 were genotyping-by-sequencing (GBS) SNP markers (Bekele *et al.* 2018; Esvelt Klos *et al.* 2016). Briefly, SNPs from the Illumina 6K chip were called initially using the Genotyping module of the GenomeStudio software v.2011.1 (Illumina Inc., San Diego, CA), followed by the elimination of multiallelic and monomorphic SNPs, as well as SNPs with poor genotype calls resulting from weak signal or ambiguous clustering through visual inspection as described in (Esvelt Klos *et al.* 2016; Tinker *et al.* 2014). GBS-SNPs were called using the UNEAK pipeline (Lu *et al.* 2013), a *de novo* GBS-SNP calling pipeline which does not require a reference genome sequence, with the *PstI‐MspI* restriction enzyme combination, as described in (Huang *et al.* 2014) with an updated nomenclature (Bekele *et al.* 2018). SNP markers of both technologies were downloaded from T3/Oat (https://oat.triticeaetoolbox.org/) and we filtered the data at per marker level with a missing rate < 0.2, heterozygosity < 0.1 and minor allele frequency (MAF) ≧ 0.05.

### Statistical analysis

All statistical analyses were performed using R (R Core Team 2018). We have tested the genotype × location effect on the original traits using the R package “lme4” (Bates *et al.* 2015) by comparing the reduced model to the full model where the genotype × location effect was included. Since the genotype× location effect was significant, subsequent analysis were performed per each location. Analysis of variance (ANOVA) was performed using the R package “augmentedRCBD” (Aravind *et al.* 2020). Broad sense heritability (*H^2^*) was estimated based on the sum of squares of the ANOVA table. Batch effect was estimated as the marginal mean of the six check varieties and the adjusted trait value for each test oat line was calculated by subtracting batch effect from initial values. Further pairwise correlation and GWA were conducted based on the adjusted values. Pairwise correlation was conducted using *cor* function in R while GWA was conducted separately for each replicate, using the method of Fixed and random model Circulating Probability Unification (FarmCPU) implemented in R (Liu *et al.* 2016). This method divides the multiple loci linear mixed model into two parts: a fixed effect model (FEM) and a random effect model (REM). FEM and REM were used iteratively. FEM includes one-by-one marker testing; multiple associated markers were further treated as covariates to control false positives. To avoid the model over-fitting problem in FEM, the associated markers, or pseudo quantitative trait nucleotide (pseudo QTN), were estimated in REM by using them to define kinship. The FEM is as follows:

$$y_{i}=M_{i1}b_{1}+M_{i2}b_{2}+\ldots +M_{it}b_{t}+S_{ij}d_{j}+e_{i}$$

where $y_{i}$ is the observation of individual i, $M_{i1},M_{i2},\ldots M_{it}$ are t pseudo QTN, $b_{1},b_{2},\ldots b_{t}$ are the corresponding effect of each pseudo QTN, $S_{ij}$ is the jth marker to be tested in the model, $d_{j}$ is its effect and $e_{i}$ is the error term following a normal distribution $N\left(0,\sigma_{e}^{2}\right).$ Initially, pseudo QTL are not available. Each marker is then tested and the most significant ones will be retained in the model and will be optimized through the REM:

$$y_{i}=u_{i}+e_{i}$$

where $y_{i}$ and $e_{i}$ is as defined in FEM and the $u_{i}$ is the total genetic effect of individual i. The expectation the individuals’ total genetic effects is zero. The variance and covariance matrix of the individuals’ total genetic effects can be modeled as $G=2K\sigma_{a}^{2}$, where K is the kinship derived from pseudo QTN and $\sigma_{a}^{2}$is an unknown genetic variance.

Bonferroni threshold at α = 0.1 was used to define pseudo QTN at FEM, which corresponded to 4.3 × 10^−6^. For each trait, zero to five PCs were included in the model to correct for the effect of population structure, and the best-fit model for a given trait was selected based on quantile-quantile plot. Given the stringency of Bonferroni correction, a *post hoc* threshold was set at a false discovery rate (FDR) of 10%. A linkage group homeologous circle diagram was created using R/circlize (Gu *et al.* 2014).

### Sequence alignment

BLASTN 2.9.0+ (Altschul *et al.* 1997) was used in a local work station to compare context sequences of trait-associated markers to the sequences of three model cereals, *Brachypodium distachyon* (strain Bd21, Brachypodium_distachyon_v3.0), maize (cv. B73, RefGen_v4), and rice (cv. Nipponbare, IRGSP-1.0). A word size of 7 was set to optimize sequence comparison between species. The E-value threshold was set at 10^−10^ due to relative short sequence of GBS markers (64 bp).

### Data availability

Phenotypic data collected in the study have been uploaded to T3/Oat: https://triticeaetoolbox.org/oat/. Supplemental files are available at FigShare. Supplementary S1 contains Figure S1 to S6: Figure S1 shows the experimental design of oat seed vigor phenotyping in this study; Figure S2 shows the growth difference between the two replicates. Figure S3 contains distribution of adjusted value of seed-vigor traits of CORE in two replicates; Figure S4 shows the correlation between traits between the two replicates; Figure S5 shows the correlation between traits in Taipei; Figure S6 shows the correlation between traits in Aberdeen. Supplementary S2 contains ANOVA results of traits. Supplementary S3 contains Manhattan plots and Q-Q plots for all traits not presented in the main body of the text. Supplementary S4 contains trait value boxplots based on allele contrast of trait-associated SNPs. Supplemental material available at figshare: https://doi.org/10.25387/g3.12721535.

## Results

### Phenotypic data

We have observed obvious growth difference between the two replicates: the replicate grown in Taipei was generally healthier than the one grown in Aberdeen (Supplementary Figure S2). A test of genotype × location interaction using mixed model on original traits showed significant genotype × location interaction (Table 2). The significant genotype× location interaction, as well as the obvious difference between the two replicates, led us to estimated adjusted genotypic values within each replicate. ANOVA was performed on traits of interest for each replicate and broad sense heritability (*H^2^*) was estimated based on the sum of squared of the ANOVA table (Supplementary S2) where ever it was possible. Root number (RN) showed significant genotypic effect (*P* < 0.001) for both replicates and its *H^2^* was high (0.86) in both replicates (Table 3). The batch effect was not significant in Aberdeen (*P* = 0.23) and was slightly significant in Taipei (*P* = 0.01; Supplementary Table). For all the other traits, batch had significant effect (*P* < 0.001) for data collected in both replicates, while genotype showed no statistically significant effect. Therefore, it was necessary to calculate adjusted value for each genotype based on the batch effect evaluated on replicated check varieties. After removing poorly grown lines and images of bad quality, the final number of individuals for root-related traits was 564 and 452 for Taipei and Aberdeen, respectively. For shoot-related traits, the final number of individuals was 567 and 592 for Taipei and Aberdeen, respectively (Table 3). All traits investigated in this study followed normal or closed-to-normal distributions (Supplementary Figure S3).

**Table 2:** Test of genotype × location interaction on original traits

Trait	Model	AIC	BIC	logLik	χ^2^	P-value	
RSA_4	Reduced	3299.1	3358.8	−1638.6			
	Full	3289.6	3354.6	−1632.8	11.6	0.000674	***
RSA_5	Reduced	3344	3403.6	−1661			
	Full	3333.2	3398.3	−1654.6	12.8	0.000344	***
SL_4	Reduced	2584.9	2643.6	−1281.4			
	Full	2568.7	2632.8	−1272.3	18.2	1.99E-05	***
SL_5	Reduced	3041.1	3100.6	−1509.5			
	Full	3019.5	3084.5	−1497.7	23.6	1.20E-06	***

Compared to the Reduced model, Full model has one additional term which is the interaction between the test genotype and location. AIC, Akaike information criterion; BIC, Bayesian information criterion; logLik, log likelihood; χ^2^, χ^2^ test value between reduced and full model with one degree of freedom; P-value, P-value of the χ^2^ test.

**Table 3:** Summary statistics of seed vigor phenotypic data

	Taipei								Aberdeen						
Trait	Min	Max	Mean	Median	SD	CV	*H^2^*		Min	Max	Mean	Median	SD	CV	*H^2^*
Root (564/452)*^a^*															
RN	2.7	4.9	3.4	3.3	0.5	13.4	0.86		2.6	5.1	3.4	3.3	0.4	13.2	0.86
RSA_3 (mm^2^)	12.1	157	93.9	95.8	24.0	25.6	0.45		NA	NA	NA	NA	NA	NA	NA
RSA_4 (mm^2^)	41	203.1	125.9	127.8	28.2	22.4	0.42		17.8	156.5	81.4	78.8	24.6	30.2	0.56
RSA_5 (mm^2^)	67.7	244.6	154.7	155.6	31.6	20.5	0.55		35	207.8	100.4	97.9	27.8	27.7	0.45
AVRSA (mm^2^)	18.1	73	45.8	45.3	9.6	21.0	0.58		7.4	56.8	30	29.4	7.9	26.3	0.45
RG_4 (mm^2^·day^-1^)	1.6	77.4	32.1	31.4	13.7	42.9	0.65		NA	NA	NA	NA	NA	NA	NA
RG_5 (mm^2^·day^-1^)	7.4	78.7	28.8	27.4	12.0	41.5	0.68		4	65.3	19	17	10.8	57.0	0.31
RRGR_4 (day^-1^)	0	2.5	0.4	0.3	0.2	65.6	0.66		NA	NA	NA	NA	NA	NA	NA
RRGR_5 (day^-1^)	0.1	1.3	0.2	0.2	0.1	50.1	0.57		0	1.2	0.3	0.2	0.2	64.4	0.32
															
Shoot (567/592)*^a^*															
SL_3 (mm)	7.2	32.99	19.3	19.1	3.7	19.2	NA		NA	NA	NA	NA	NA	NA	NA
SL_4 (mm)	13.43	73.37	33.9	33.2	6.5	19.2	0.71		9.7	38.4	20.2	20.1	4.9	24.3	NA
SL_5 (mm)	16.43	108.98	54	52.7	13.3	24.6	0.71		14.3	66.3	32.7	32	7.8	23.9	NA
SG_4 (mm·day^-1^)	1.32	43	14.6	14.3	4.4	30.0	NA		NA	NA	NA	NA	NA	NA	NA
SG_5 (mm·day^-1^)	0.67	45.88	20.2	19.5	8.3	41.2	0.72		2.1	31.4	12.5	12.1	4.5	36.1	NA
SRGR_4 (day^-1^)	0.08	1.87	0.8	0.8	0.2	31	NA		NA	NA	NA	NA	NA	NA	NA
SRGR_5 (day^-1^)	0.04	1.26	0.6	0.6	0.2	33.5	0.75		0.1	3.3	0.7	0.6	0.2	36.8	NA

a No. of individual of good quality data collected in Taipei (left to the slash) and in Aberdeen (right to the slash).

NA, not available. Broad sense heritability (*H^2^*) are not available when the data of some check varieties were missing in some blocks, impeding the variance estimation.

In general, higher values were observed for data collected in Taipei than in Aberdeen, except for shoot relative growth rate for day 5 (SSGR5, Table 3). *H^2^* followed similar trend which was generally higher in the data collected in Taipei than in Aberdeen, except for RN and RSA_4 (Table 3). Root traits generally showed moderate *H^2^* (0.31 to 0.68, Table 3) while *H^2^* for shoot traits were more important (0.71 to 0.75, Table 3). RN was stable and highly correlated across the two replicates (Pearson’s *r* = 0.69, *P* < 0.001, Supplementary Figure S4): RN varied between 2.7 and 4.9 in Taipei and 2.6 and 5.1 in Aberdeen while the average (3.4) and the median (3.3) were identical for both replicates (Table 3). Other original traits, *i.e.*, RSA and SL, were highly correlated across different days within replicate (Pearson’s *r* = 0.77 – 0.93, *P* < 0.001, Supplementary Figures S5 and S6), while the correlation was significant, but to a lesser extent, for the joint consideration of both replicates (Pearson’s *r* = 0.31 to 0.38, *P* < 0.001, Supplementary Figure S4). A similar trend was observed for the derived traits.

### Genome-wide association and homeolog inference

**Figure 2:**
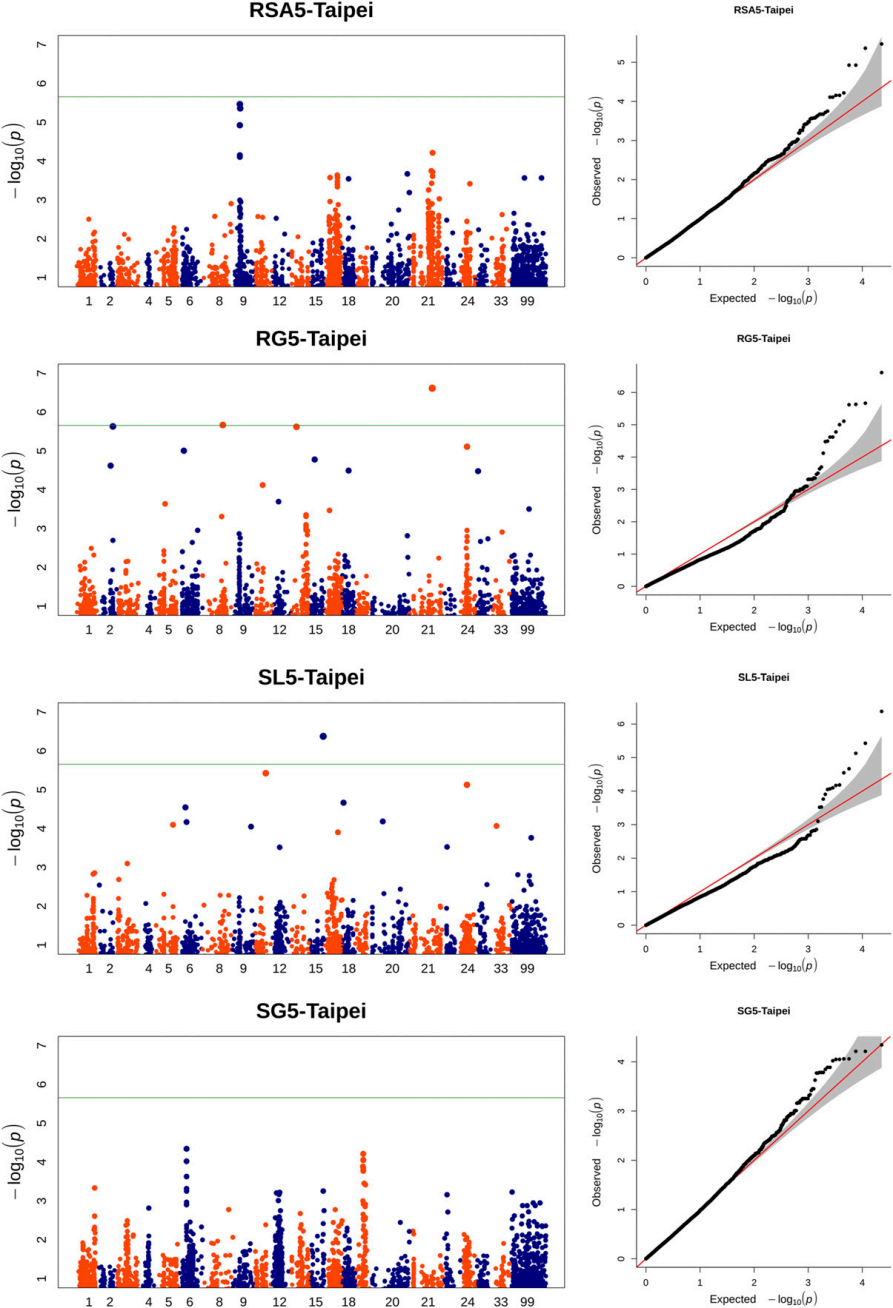
Manhattan and quantile-quantile plots for RSA5, RG5, SL5, SG5 collected in Taipei. Green horizontal line indicates the Bonferroni threshold at α=0.1.

We have identified the most appropriate GWA model for each trait based on quantile-quantile plots (Figure 2, Supplementary S3). We have identified 34 and 16 unique loci associated with root traits and shoot traits, respectively, which corresponded to 41 and 16 unique SNPs (Table 4 and Table 5, FDR < 10%), respectively. A unique locus was defined as a unique position on the oat consensus map, which may harbor several SNPs. MAF of trait-associated SNPs varied between 0.06 and 0.49 and the allelic substitution effect were generally small, even for highly significant SNPs (Table 4 and Table 5). For root traits, the 41 SNPs were associated with RSA_5, RG_4, RG_5, RRGR_4, RRGR_5 and AVRSA, which were allocated on 16 linkage groups (LG) while 2 SNPs were unmapped; Seven of them were highly significant, showing a *p*-value below the Bonferroni threshold. SNPs avgbs_29471.1.50 and avgbs_cluster_33692.1.63 were associated with more than one trait, both with highly correlated RSA_5 and AVRSA.

**Table 4:** Root trait-associated markers identified in the CORE panel

Trait	Marker	Mrg*^a^*	Pos (cM)	Sub. effect^b^	*P*-value^c^	MAF	Literature^d^
RSA_5	avgbs_29471.1.50	9	44.7	−7.0	3.40E-06	0.35	
	avgbs_cluster_34557.1.39	9	44.7	−6.5	1.19E-05	0.38	
	avgbs2_169620.1.39	9	44.7	−6.5	1.19E-05	0.38	
	avgbs_cluster_33692.1.63	9	47.1	−6.8	4.36E-06	0.39	
RG_4	GMI_ES01_c5178_479	4	45.0	2.2	2.47E-05	0.20	
	avgbs_115262.1.42	5	114.7	−4.0	5.17E-08*	0.06	
	avgbs2_46371.1.64	5	131.6	6.3	2.12E-05	0.06	
	avgbs2_46371.1.8	5	131.6	6.3	2.12E-05	0.06	
	avgbs_12458.1.21	9	29.6	−2.8	2.33E-06	0.31	
	avgbs_205346.1.41	12	57.4	3.0	6.68E-08*	0.36	1
	avgbs_cluster_34786.1.14	17	87.1	3.1	3.86E-08*	0.19	
	avgbs_218823	18	10.9	−1.6	1.50E-05	0.38	
	avgbs_cluster_38064.1.46	18	12.8	−2.4	2.98E-05	0.08	
	avgbs_cluster_37097.2.27	23	22.3	−2.1	4.49E-05	0.29	
	avgbs2_102913.1.47	28	57.3	−3.4	2.43E-05	0.09	3, 4
	avgbs2_10058.2.59	UKN	—	4.9	2.51E-06	0.06	
RG_5	avgbs_cluster_26155.1.55	2	72.7	2.6	2.40E-05	0.37	2
	avgbs2_122749.1.55	2	72.7	2.6	2.40E-05	0.37	2
	avgbs_cluster_35609.1.46	2	87.3	−1.3	2.33E-06	0.4	
	avgbs_49723.1.14	8	129.6	2.6	2.15E-06*	0.29	6
	avgbs_21913.1.62	13	35.9	2.3	2.39E-06	0.35	
	avgbs_cluster_41284.1.50	15	32.7	−1.9	1.66E-05	0.42	
	avgbs_92060.1.20	18	40.0	−1.8	3.21E-05	0.31	2
	avgbs_cluster_25488.1.14	21	146.0	−3.1	2.42E-07*	0.16	5
	avgbs_120739.1.46	24	41.8	−2.8	7.76E-06	0.09	
	avgbs_218324	28	17.1	−2.6	3.32E-05	0.07	3
RRGR_4	GMI_DS_LB_8372	13	59.6	−0.047	8.46E-07*	0.27	
	avgbs_cluster_15604.1.25	17	55.6	−0.055	2.38E-05	0.09	
	avgbs2_53658.1.10	18	60.3	−0.104	1.26E-08*	0.08	
	avgbs_56897.1.18	28	36.8	−0.086	3.51E-05	0.18	
	avgbs_cluster_31411.1.54	28	43.8	−0.088	5.36E-08*	0.17	4
	avgbs_cluster_29960.1.24	28	43.8	0.044	1.03E-05	0.15	4
	GMI_GBS_13776	28	43.8	−0.084	3.06E-05	0.19	4
	GMI_GBS_96525	28	43.8	−0.085	2.89E-05	0.19	4
RRGR_5	avgbs_cluster_10425.1.7	1	57.2	0.019	6.77E-06	0.46	
	avgbs_200217	4	50.3	0.021	9.28E-07*	0.34	
	avgbs_cluster_10309.1.10	6	28.1	0.021	3.43E-07*	0.44	2
	avgbs_cluster_10035.1.11	13	106.5	0.020	1.84E-05	0.22	
	avgbs_207710	UKN	—	−0.029	1.41E-06*	0.1	
AVRSA	avgbs_29471.1.50	9	44.7	−2.1	3.00E-06	0.35	
	avgbs_cluster_33692.1.63	9	47.1	−1.9	1.17E-05	0.39	
	avgbs_cluster_28147.1.55	17	84.3	1.9	9.04E-06	0.46	
	GMI_ES03_c19505_223	17	85.3	1.9	6.90E-06	0.49	

a : “UKN” for unmapped SNPs. ^b^: allelic substitution effect. ^c^:* indicated markers having *P-value* below Bonferroni threshold. ^d^: 1, (Holland *et al.* 1997); 2, (Huang *et al.* 2020); 3, (Siripoonwiwat *et al.* 1996); 4, (Sunstrum *et al.* 2019); 5, (Wooten *et al.* 2009); 6, (Tumino *et al.* 2017).

**Table 5:** Shoot trait-associated markers identified in the CORE panel

Trait	Marker	Mrg*^a^*	Pos (cM)	Sub. effect^b^	*P*-value^c^	MAF	Literature^d^
SL_5	avgbs_cluster_3321.1.50	11	69.3	−2.8	3.75E-06	0.18	
	avgbs_cluster_19949.1.14	15	87.2	−2.5	4.19E-07*	0.44	
	avgbs2_169934.1.10	24	41.3	−3.1	7.46E-06	0.12	
SRGR_5	GMI_ES17_c1687_437†	1	39.3	−0.058	1.82E-05	0.15	
	avgbs_22768	2	27.3	0.039	6.43E-07*	0.23	8
	avgbs_cluster_38112.1.47	3	55.8	−0.035	3.20E-06	0.34	
	avgbs_cluster_49442.1.14†	3	77.8	−0.061	3.82E-06	0.12	
	GMI_ES03_c5596_272	3	101.6	0.027	2.96E-06	0.43	2
	avgbs_cluster_1872.1.10	4	40.2	−0.027	3.43E-05	0.44	
	avgbs2_96578.1.61†	9	78.3	−0.085	4.38E-06	0.15	
	avgbs_125970.1.51	11	42	0.029	4.79E-06	0.41	
	avgbs_cluster_3597.1.11†	12	33.4	−0.062	3.28E-08*	0.25	4,7
	avgbs2_183559.1.25	12	40.2	−0.047	3.49E-07*	0.19	1,3,4,6
	avgbs_cluster_19949.1.14	15	87.2	−0.034	1.53E-07*	0.44	
	avgbs_cluster_26077.1.22	20	226.6	−0.022	2.57E-05	0.44	
	avgbs_220709.1.57	21	22.7	−0.042	1.52E-06*	0.21	4
	avgbs_310061†	UKN	—	−0.122	3.36E-07*	0.25	

a : “UKN” for unmapped SNPs. ^b^: allelic substitution effect. ^c^:* indicated markers having *P-value* below Bonferroni threshold. ^d^: 1, (Holland *et al.* 1997); 2, (Huang *et al.* 2020); 3, (Siripoonwiwat *et al.* 1996); 4, (Sunstrum *et al.* 2019); 5, (Wooten *et al.* 2009); 6, (Beer *et al.* 1997); 7, (Herrmann *et al.* 2014); 8, (Zimmer *et al.* 2018). †: associated SNPs identified on Aberdeen replicate.

The 16 SNPs associated with shoot related-trait were distributed on 10 LG, with one unmapped. Seven of these SNP were statistically significant at the Bonferroni threshold. For shoot trait-related SNPs, avgbs_cluster_19949.1.14 was associated with both SL_5 and SRGR_5 and the two SNPs associated with SG_5, avgbs_cluster_38112.1.47 and avgbs_cluster_1872.1.10, were also significantly associated with SRGR_5.

**Figure 3:**
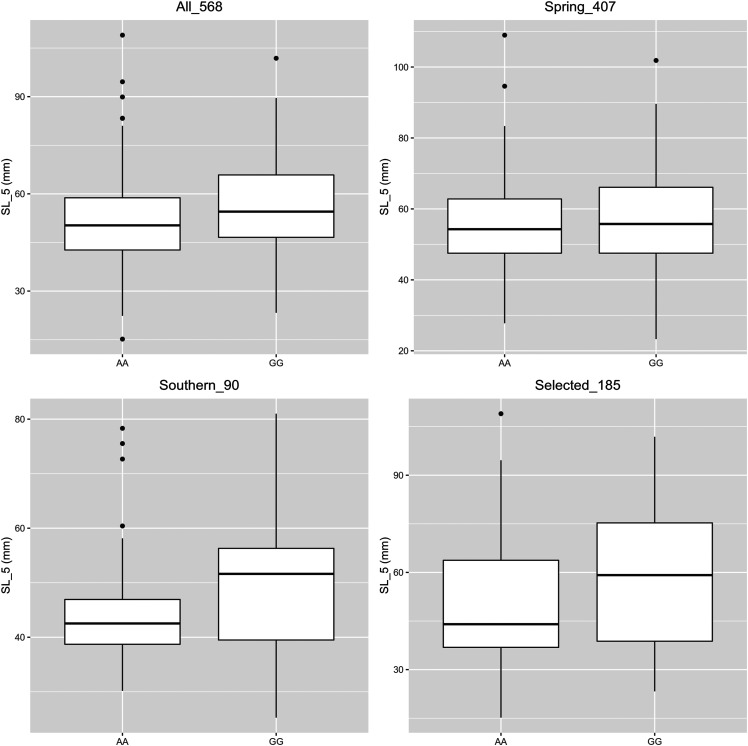
Allelic contrast of avgbs_cluster_19949.1.14 in the whole CORE panel (568 individuals), a subset of spring oats from the CORE (407 individuals), a subset of Southern oats from the CORE (90 individuals) and the top and bottom 10% selected 185 individuals from CORE.

In order to have a clearer idea about the allele effect of the trait-associated markers, we have examined the phenotypic distribution of different alleles for markers associated with original traits, as well as markers identified using Bonferroni threshold for the derived traits (Supplementary S4). The median of phenotypic value between the two alleles did not differ much, which was concordant with the weak substitution effect of the trait-associated markers (Table 4 and Table 5). Meanwhile, when we looked into the allelic mean within germplasm of different genetic background, *i.e.*, spring *vs.*. Southern oats, we can notice a more pronounced allelic contrast in one genetic background than the other. For example, the allelic contrast of the marker avgbs_cluster_19949.1.14, associated with SL_5 collected in Taipei, was more pronounced in Southern germplasm than in the spring germplasm (Figure 3). When we selected the top and bottom 10% of individual based on SL_5, the allelic contrast of this marker was more accentuated than the full CORE panel, implying this marker, although showing weak effect on the full panel, may contribute more to the vigor difference between specific genetic materials.

**Figure 4:**
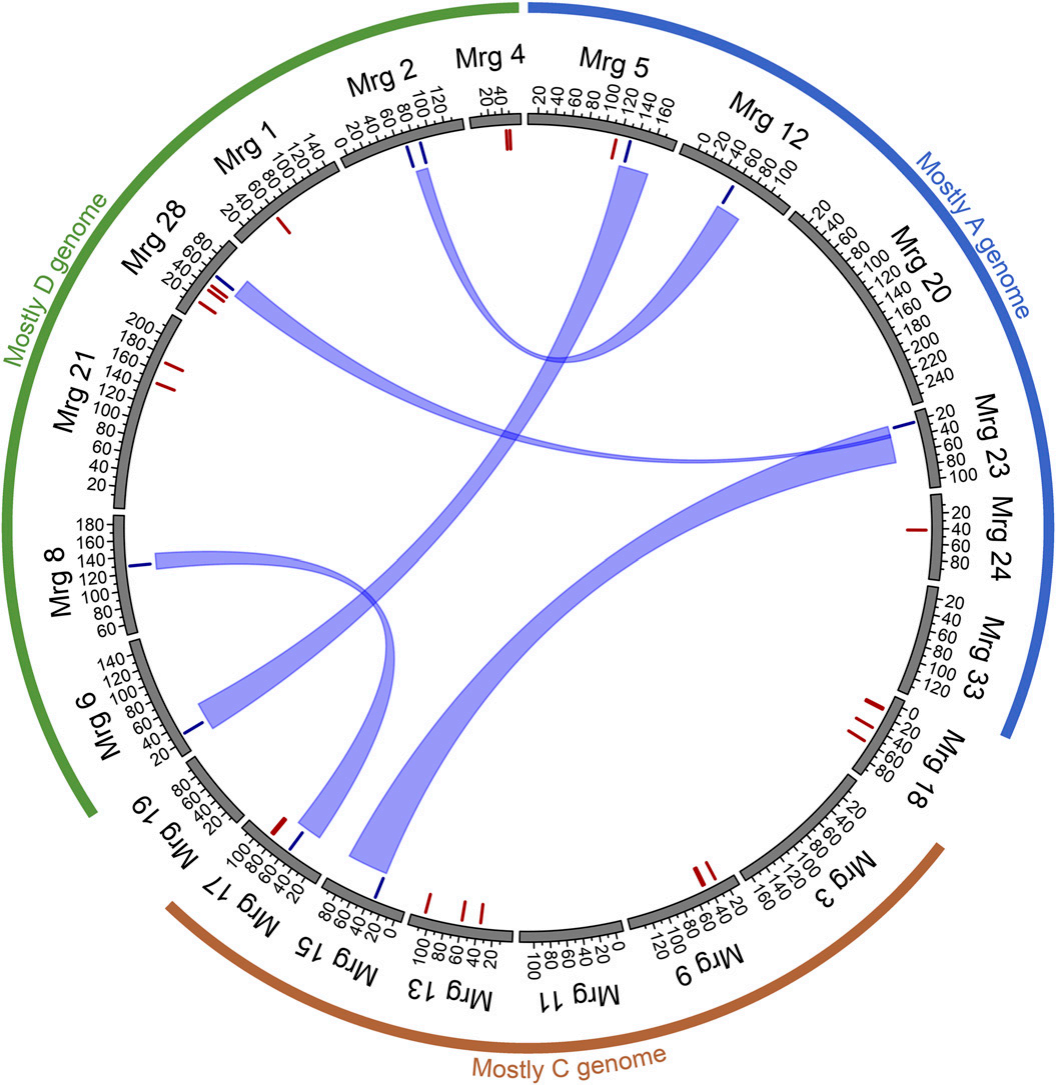
Root-trait-associated markers positioned on the oat consensus map. Blue lines at the inner circle indicate trait-associated markers located within homeologous regions while red lines represented trait-associated markers not located within homeologous regions. Homeologous regions are related by the blue ribbon. The inferred homeologous regions were from Chaffin *et al.* (2016) and the annotations of oat subgenomes were based on Yan *et al.* (2016a).

**Figure 5:**
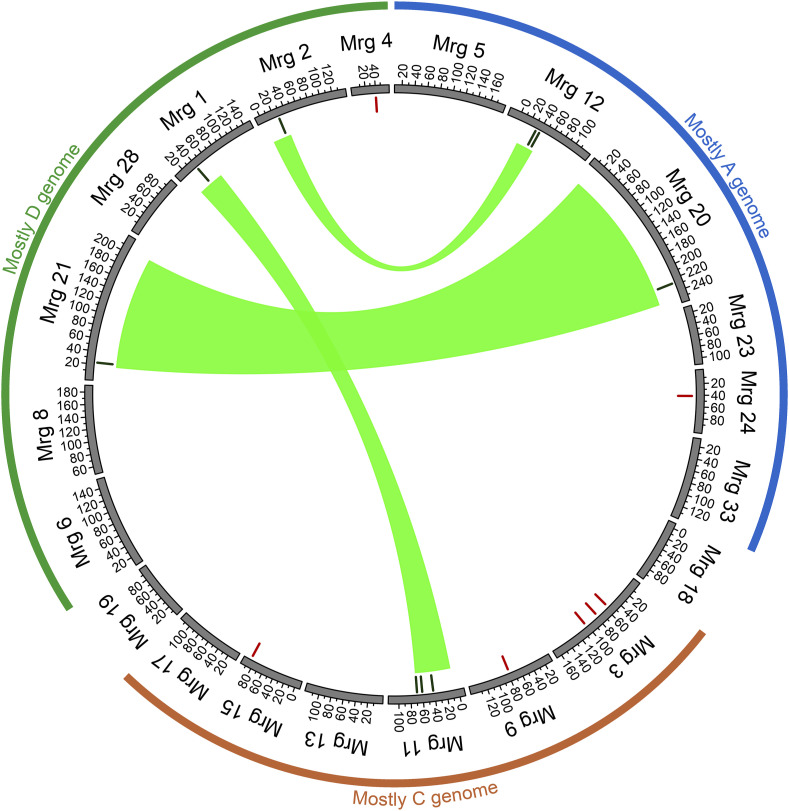
Shoot-trait-associated markers positioned on the oat consensus map. Blue lines at the inner circle indicate trait-associated markers located within homeologous regions while red lines represent trait-associated markers not located within homeologous regions. Homeologous regions are related by the blue ribbon. The inferred homeologous regions were from Chaffin *et al.* (2016) and the annotations of oat subgenomes were based on Yan *et al.* (2016a).

We further investigated the homeologous relationship between associated SNPs based on the homeologous regions inferred by Chaffin *et al.* (2016). For root traits, nine loci were organized into four sets of homeologous regions, comprising of [Mrg02, Mrg12], [Mrg05, Mrg06], [Mrg08, Mrg17], and [Mrg15, Mrg23, Mrg28] (Figure 4). For shoot-related traits, nine loci were organized into three sets of homeologous regions, consisted of [Mrg01, Mrg11], [Mrg02, Mrg12], and [Mrg20, Mrg21] (Figure 5).

Most of the significant associations were identified on measurements collected in Taipei except for five significant SNPs associations with shoot traits collected in Aberdeen (GMI_ES17_c1687_437, avgbs_cluster_49442.1.14, avgbs2_96578.1.61, avgbs_cluster_3597.1.11, avgbs_310061; Table 5). Nonetheless, SNPs associated to traits collected from two replicates fell into the homeologous regions [Mrg01, Mrg11] and [Mrg02, Mrg12].

### Identification of candidate genes

To identify candidate genes related to the seed vigor-related SNPs identified in this study, we blasted the context sequences around the SNPs to the reference genome of three model monocots, brachypodium, maize and rice (Table 6). Four SNPs were highly similar to sequences from all three organisms, except avgbs_29471.1.50 which matched only rice and maize. All significant matches (*E* < 10^−10^) were retained. Context sequence around avgbs_cluster_41284.1.50, associated to RG_5, was matched to *Os01g0675500*, *Bradi2g46410*, and *Zm00001d043879* which were predicted to be similar to a glucuronosyltransferase, the SNP (A/T) would change phenylalanine to isoleucine. Context sequence around GMI_ES17_c1687_437, associated with SRGR_5, was matched to *Os03g0191100*, *Bradi1g71800*, *Zm00001d048218*, and *Zm00001d028008*, which was predicted to be similar to a mitochondrial carrier protein domain containing protein in rice. The SNP (A/G) was located in the coding region and was a synonymous mutation. Context sequence around avgbs2_183559.1.25, also associated with SRGR_5, was matched to *Os06g0146400d*, *Bradi1g51010.1d*, and *Zm00001d036145*, which was predicted to code for an iron-sulfur cluster protein in rice and brachypodium. The SNP (T/C) was located in the coding region and would cause a change from leucine to proline. Context sequence around avgbs_29471.1.50, associated with AVRSA, matched to genome sequence of rice and maize, although no known gene model was predicted for the matched sequences.

**Table 6:** Candidate genes for oat seed vigor-related traits

		Rice				Brachypodium				Maize			
Marker*^a^*	Trait	Chr.	E value	Query recovery	Gene model	Chr.	E value	Query recovery	Gene model	Chr.	E value	Query recovery	Gene model
avgbs_cluster_41284.1.50	RG_5	1	9.00E-15	100%	*Os01g0675500*^b^	2	6.00E-20	100%	*Bradi2g46410*^b^	8	1.00E-13	96%	
										3	3.00E-11	100%	*Zm00001d043879*^b^
avgbs_29471.1.50	AVRSA	12	3.00E-22	96%		—	—	—	—	3	3.00E-22	96%	
										9	3.00E-22	96%	
										2	3.00E-20	96%	
GMI_ES17_c1687_437	SRGR_5	3	1.00E-36	100%	*Os03g0191100*^c^	1	2.00E-31	100%	*Bradi1g71800*	9	1.00E-17	100%	*Zm00001d048218*
										1	2.00E-12	100%	*Zm00001d028008*
										4	2.00E-12	100%	
avgbs2_183559.1.25	SRGR_5	6	3.00E-21	100%	*Os06g0146400*^d^	1	3.00E-17	98%	*Bradi1g51010.1*^d^	6	2.00E-14	100%	*Zm00001d036145*
										9	9.00E-14	89%	

a Context sequences used for BLAST were 64 bp long for avgbs- series of SNPs, and 122 bp for GMI_ES17_c1687_437. ^b^similar to glucuronosyltransferase. ^c^ similar to mitochondrial carrier protein; ^d^ predicted to code for iron-sulfur cluster protein.

## Discussion

### Seed vigor phenotyping pipeline

Measuring root traits has long been difficult and laborious, especially if many accessions are to be assayed. In this study, we used a simple approach with germination pouches which allowed us to measure seed vigor-related traits on a large population of 650 individuals. Previous studies on seed or seedling vigor regarding root traits have used other methods, such as growing plants in paper rolls or hydroponic systems, with images acquired through scanning (Atkinson *et al.* 2015; Clark *et al.* 2013; Pace *et al.* 2015). Compared to paper rolls or hydroponic systems, germination pouches occupy much less space and are more suitable for large-scale phenotyping. Germination pouches also provide a non-destructive measurement at multiple time points, and image quality can be effectively controlled once the appropriate camera parameters are identified. Image acquisition was time-efficient in our study: pictures of 150 oat lines were taken within less than 2 hr. The maximum observation time was conditioned by the size of the germination pouch and the growth rate of the target plant. In our experiment, the roots of fast-growing oat lines would reach the bottom limit of the germination pouch after five days under 25°, therefore, we limited our experiment to five days. Despite this limitation, another advantage of the germination pouch is its flexibility. With slight changes to the number of seeds per pouch, more traits, such as root angle, root depth and root diameter, could be measured.

The graphical user interface of the Fiji image analysis software facilitated flexible analysis adjustment and batch image processing which enabled a speedy and flexible image analysis. We opted to measure total root surface area (RSA) instead of the more commonly used root length to characterize seedling root growth. Measurement of root surface area, potentially more reflective of root vigor, was made possible by the imaging system. This side-stepped difficulty with tangled or intertwined roots that can complicate root length measurement, even with image analysis tools. In addition, previous studies have shown high correlation between RSA and total root length (Pace *et al.* 2015; Wang *et al.* 2018). Therefore, we consider our study to be comparable to previous work.

### Seed vigor variation

Seed vigor is a complex trait involving shoot and root growth. In this study, variation among lines was observed for all traits in both replicates with a variable *H^2^*. The data collected in Taipei were generally larger than the data collected in Aberdeen (Table 3). Obvious growth difference was observed on the seedling pictures taken at both locations (Supplementary Figure S2). This should be related to the sensitivity of seed vigor vis-à-vis of environmental factors. Indeed, we have observed that roots would shrivel at the contact of the germination pouch and we observed a higher proportion of shriveled roots in the Aberdeen replicate. There may be other unknown environmental factors influencing on the phenotyping pipeline. For example, unknown environmental effects on coleoptile length in wheat have been noticed under controlled environments (Spielmeyer *et al.* 2007). In rice, mesocotyl length, an integral part of seedling shoot length, is influenced by factors like light, auxin, abscisic acid, jasmonate, and strigolactone (Hu *et al.* 2013; Tsunoda and Takahashi 1984). Roots respond to environmental factors like nutrients, water availability, and plant hormones such as auxin and cytokinin; previous studies have identified large genotype × environment and QTL × environment interactions in rice (MacMillan *et al.* 2006; Kamoshita *et al.* 2002). In our study, we have observed a lower *H^2^* for root-related traits than that for shoot-related traits, which suggested the higher sensitivity of roots to the environmental factors.

Root number was relatively stable across the two replicates, compared to other seed vigor traits. Root number may be a marker of crop improvement since it has been reported that an increased number of seminal roots was observed in domesticated wheat, maize and barley compared to their wild relatives (Robertson *et al.* 1979; Burton *et al.* 2013; Grando and Ceccarelli 1995). In wheat, although both wild and domesticated materials possessed five root primordia in the embryo, the 4^th^ and 5^th^ primordia of wild wheat would not be activated unless the plant sensed water stress (Golan *et al.* 2018). Under well-watered condition, three seminal roots were sufficient to maintain the seedling growth. Such information is not yet available for oats. Despite the high *H^2^* for RN, we did not identify any marker associated with RN. It is mostly probably that our germplasm consisted of elite germplasm where the RN did not vary much (Table 3). We may be able to find more RN variation in landraces or wild relatives, as shown in a wheat study (Golan *et al.* 2018).

### Genetic architecture of oat seed vigor and candidate genes

Seed vigor has been shown to be controlled by multiple QTL in wheat, maize and rice (Pace *et al.* 2015; Atkinson *et al.* 2015; Rebetzke *et al.* 2014; Li *et al.* 2017; Lu *et al.* 2016; Wang *et al.* 2018). We applied GWA to dissect the genetic architecture of seed vigor in elite oat lines. To avoid the possible type II error, *i.e.*, consider a marker-trait association negative while it is indeed positive, due to the stringency of Bonferroni correction, we also used FDR to identify significant association. Trait-associated SNPs identified in our study showed small allelic substitution effect (Table 4 and Table 5), together with the moderate *H^2^* of our trait (Table 3) suggested that seed vigor in oat was complex and controlled by many loci of minor effect. Although SNPs significantly associated with data collected in Taipei were not those associated with data collected in Aberdeen, all significant markers were organized into homeologous regions. This both strengthens our confidence in these associations and suggests that conserved regions from different subgenomes might influence these similar traits. Ten loci identified in our study overlapped with, or were close to (< 5 cM), previously reported plant height QTL (Holland *et al.* 1997; Huang *et al.* 2020; Siripoonwiwat *et al.* 1996; Sunstrum *et al.* 2019; Wooten *et al.* 2009; Beer *et al.* 1997; Herrmann *et al.* 2014)., providing further support for these QTL. We have focused on plant height QTL in the literature because the information on oat seedling QTL is lacking and the positive correlation between root size and plant height has been revealed in field studies for oats (Li *et al.* 2008; Zhang *et al.* 2009).

It is known that trait-associated SNP identified using GWA may not be directly the causal SNPs but in linkage disequilibrium with the causal SNPs (Faye *et al.* 2013; Schaid *et al.* 2018). Nevertheless, we tried to investigate whether trait-associated SNPs identified in the present study were located in plausible candidate genes for seed vigor in rice, brachypodium and maize. The context sequence of avgbs_cluster_41284.1.50, associated to RSA_5, showed great similarity with genes predicted to code for a glucuronosyltransferase in rice, brachypodium and maize. The gene model identified in rice, *Os01g0675500*, belonged to OsGT43 family which has been shown to be involved in xylan elongation (Lee *et al.* 2014). Homologs of GT43 family in *Arabidopsis* and *Brachypodium* have been reported to be essential for xylan biosynthesis, a major component of cell wall, required during root development (Lee *et al.* 2010; Wu *et al.* 2010; Whitehead *et al.* 2018). In addition, its maize ortholog, *Zm00001d043879*, exhibited high expression level in primary root and root cortex (Walley *et al.* 2016). Therefore, this is a plausible candidate gene for oat early seed vigor. GMI_ES17_c1687_437 matched to a predicted mitochondrial carrier protein. At germination, seeds exit the extreme quiescent state and enter into an active state upon water uptake, which activates seed metabolism and the biogenesis of mitochondria. It has been shown that a mitochondrial carrier family is involved in arginine metabolism during rice seed germination (Taylor *et al.* 2010). On the other hand, the maize gene model, *Zm00001d048218*, showing the highest similarity with GMI_ES17_c1687_437, exhibited high expression in the primary root and coleoptile of germinating seeds (Stelpflug *et al.* 2016; Hoopes *et al.* 2019), which together suggested this candidate apropos to seed vigor. The context sequence of avgbs2_183559.1.25, associated with SRGR_5, showed similarity to *Os06g0146400*, *Bradi1g51010*, and *Zm00001d036145*. Gene prediction in rice and brachypodium suggested the gene coding for iron-sulfur cluster protein. Iron-sulfur cluster proteins are integral to plant growth and development. Defects in some proteins involved in iron-sulfur assembly and transfer cause delayed growth or lethal seedling in *Arabidopsis* (Lu 2018). The maize ortholog *Zm00001d036145* has no available function prediction and its expression in maize seedling was weak (Stelpflug *et al.* 2016; Hoopes *et al.* 2019).

In addition to the investigation on plausible seed vigor candidate genes, the homeologous relationship between trait-associated-SNPs supports the reliability of our results. Indeed, for polyploidy crops, gene families tend to organzed in homeologous regons as a consequence the polyploidization. It has been shown in cotton that the two homeologous copies of *GhSusA1*, a gene increased cotton fiber yield and quality, were both expressed in ovules and fibers at different days post-anthesis (Jiang *et al.* 2012). The perspective of making use of homeologous relationship to identify candidate genes/loci for crop improvement has recently been reiterate in a review article (Rasheed *et al.* 2020).

## Conclusion

The image-based phenotyping pipeline enabled us to measure seed vigor traits across a large oat panel in an efficient way. We observed variation within the traits that we have investigated. The moderate *H^2^*, together with small effect trait-associated markers, suggested that seed vigor in oat is complex and under control of many loci of small effect. To make full use of data collected in the present study, further experiment in field condition should complement the results of the present study which were collected in lab condition. It will be interesting to explore the trait variation and its underlying genetics in other genetic background, such as landraces, or further study the physiological implications using contrast genotypes. Fifty loci were significantly associated with root or shoot traits and some of them were located at inferred homeologous regions. We further identified four SNP markers located within candidate gene models among which are highly plausible biological candidate genes for seed vigor: a glucuronosyltransferase involved in xylan synthesis (avgbs_cluster_41284.1.50), mitochondrial carrier protein (GMI_ES17_c1687_437), and iron-sulfur protein (avgbs2_183559.1.25). We have provided a first large GWA study in oat seed vigor within elite germplasm under mild temperature.
